# Role of LncRNAs in regulating cancer amino acid metabolism

**DOI:** 10.1186/s12935-021-01926-8

**Published:** 2021-04-13

**Authors:** Yuhong Guo, Bin Lv, Renfeng Liu, Zhengzai Dai, Feifei Zhang, Yiping Liang, Bo Yu, Duo Zeng, Xiao-Bin Lv, Zhiping Zhang

**Affiliations:** 1 Jiangxi Key Laboratory of Cancer Metastasis and Precision Treatment, The Third Affiliated Hospital of Nanchang University, Northern 128 Xiangshan Road, Nanchang, 330008 Jiangxi People’s Republic of China; 2 Department of Orthopedics, The Third Affiliated Hospital of Nanchang University, Northern 128 Xiangshan Road, Nanchang, 330008 Jiangxi People’s Republic of China; 3 Nanchang Key Laboratory of Orthopaedics, The Third Affiliated Hospital of Nanchang University, Nanchang, China; 4 Medical Department of Graduate School, Nanchang University, Nanchang, 330006 Jiangxi China

**Keywords:** lncRNA, Metabolism, Amino acid, Glutamine, Cancer

## Abstract

The metabolic change of tumor cells is an extremely complicated process that involves the intersection and integration of various signal pathways. Compared with normal tissues, cancer cells show distinguished metabolic characteristics called metabolic reprogramming, which has been considered as a sign of cancer occurrence. With the deepening of tumor research in recent years, people gradually found that amino acid metabolism played crucial roles in cancer progression. Long non-coding RNAs (lncRNAs), which are implicated in many important biological processes, were firstly discovered dysregulating in cancer tissues and participating in extensive regulation of tumorigenesis. This review focuses on the reprogramming of amino acid metabolism in cancers and how lncRNAs participate in the regulatory network by interacting with other macromolecular substances. Understanding the functions of lncRNA in amino acid reprogramming in tumors might provide a new vision on the mechanisms of tumorigenesis and the development of new approaches for cancer therapy.

## Background

The proliferation of normal cells requires the continuous accumulation of substances, so as to produce offspring cells. The accumulated substances in cells include proteins, lipids and nucleic acids, which are essential for cell proliferation [1]. In the past three decades, the studies on oncogenes revealed that the characteristic phenotype of cancer cells is often caused by somatic mutations, and it is the integration of these mutations that lead to the changes of various signal pathways and the state of cell metabolism. The PI3K/AKT/mTOR and the AMP-activated protein kinase pathways are more common dysregulated pathways in cancer [2, 3]. Researches on tumorigenesis have made it clear that these pivotal oncogenic signals lead to the unique metabolic characteristics of tumor cells and support their proliferation. Therefore, the change of cell metabolism is considered as a crucial hallmark in the development of cancer [4].

The occurrence of cancer often depends on the reprogramming of cell metabolism, and the metabolism reprogramming enables tumor cells to obtain the necessary nutrients from the nutrition deficient environment. Generally speaking, tumor cells metabolize glucose, fatty acids and glutamine at a much higher rate than normal cells [5]. Metabolism reprogramming is regarded as a cancer-specific characteristic, including dysregulation of glucose and glutamine metabolism, changes in lipid biosynthesis and decomposition, and so on [6, 7]. In order to maintain their proliferation, tumor cells need to increase ATP production, synthesize macromolecules and reduce the generation of reducing substances or other metabolic auxiliary materials [8]. Besides, cancer cells must adapt their metabolism to the dynamic changes in the process of tumor development, so as to maintain the energy level, redox status, cellular signaling and biosynthesis, thus promoting tumor growth [9]. In normal tissues, cells tend to generate energy through oxidative phosphorylation by the mitochondria, and in the absence of oxygen, glucose was catabolized to lactic acid through glycolysis. Interestingly, even in the presence of sufficient oxygen, tumor cells still use the glycolysis pathway to generate energy, in order to meet the rapid proliferation of cancer cells [10]. This process is known as the Warburg Effect. Currently, the Warburg effect has been observed in various types of tumors and has been widely accepted as a symbol of metabolic changes in cancer cells [10]. Since the discovery of the Warburg effect, the majority of researches about tumor metabolism have focused on glucose metabolism. Recently, with the deepening of the research, people are aware that other nutrients, such as amino acids, for the process of tumor cell metabolism play a key role [11].

### LncRNAs: a brief introduction

**Fig. 1:**
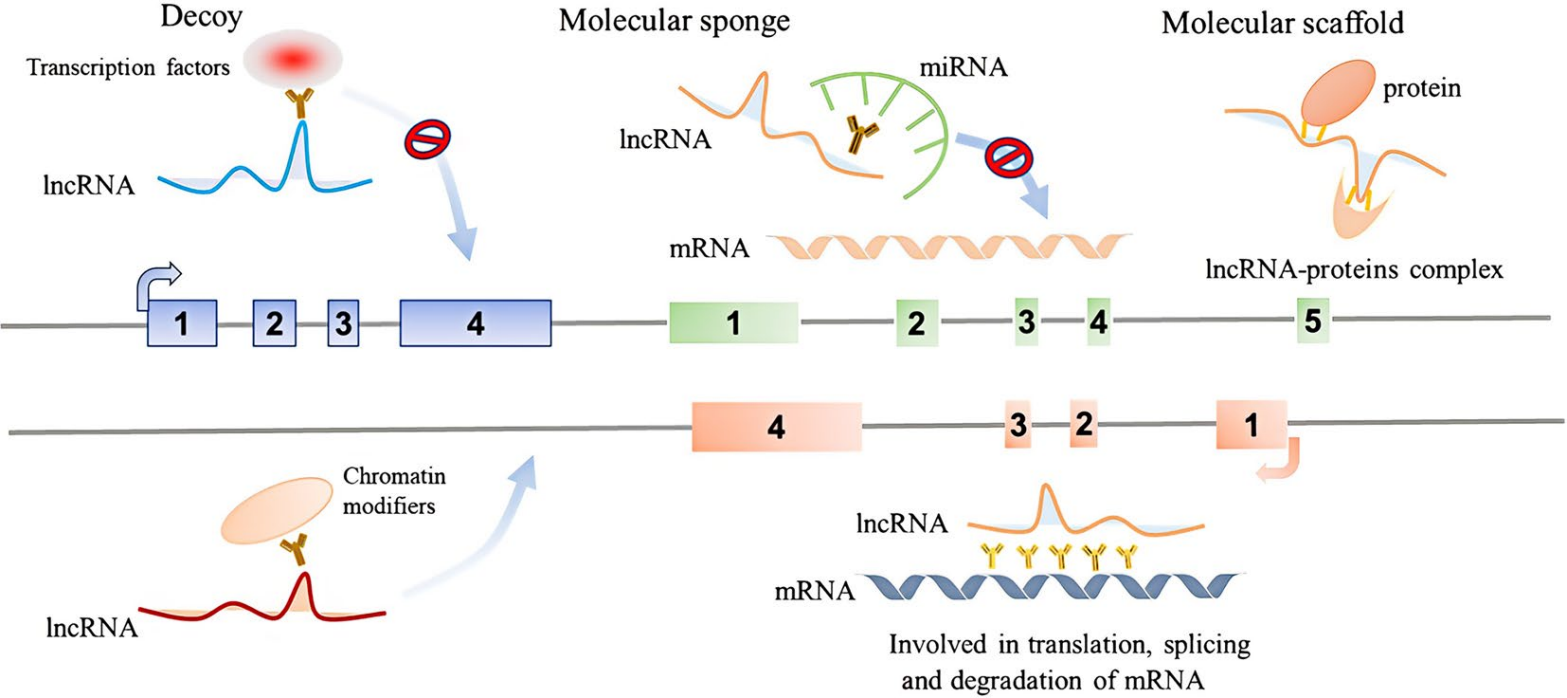
Typical molecular mechanisms of lncRNA

**Fig. 2:**
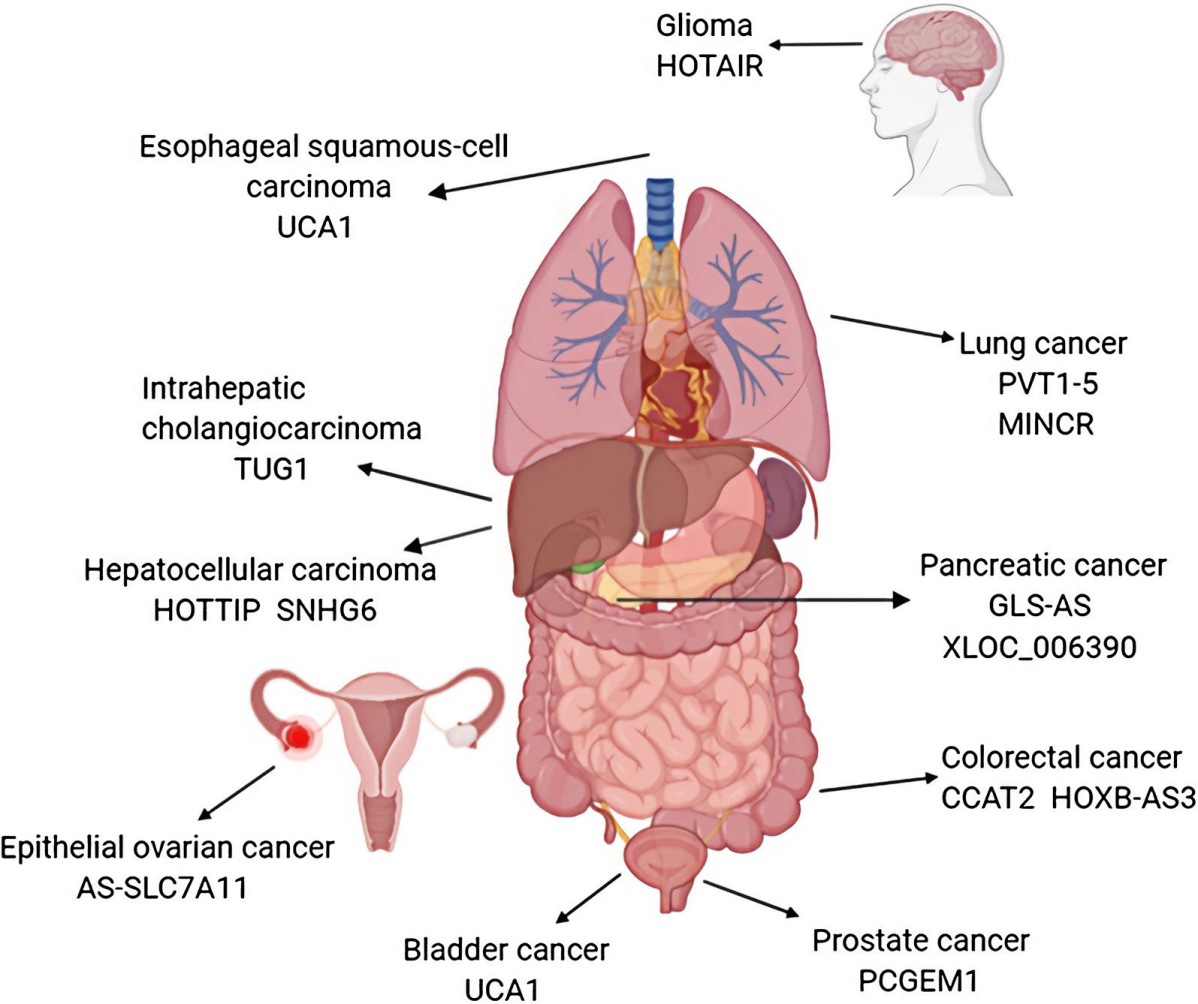
Amino acid metabolism involving lncRNAs that are dysregulated in cancer

Eukaryotes produce many types of RNAs, which play a crucial role in the transmission of genetic information and often exist in specific subcellular localization. The synthesis, processing and transportation of RNA are closely involved in the regulation of cell functions [12]. Less than 2 % of the human genome encodes protein-coding RNA, and more than 90 % of the total genome is transcribed into noncoding RNA(ncRNA) [13]. LncRNA is a kind of RNA transcript with more than 200 nucleotides, which has little protein-coding potential. It is usually transcribed by RNA polymerase II with 5’-end cap, polyadenylation and splicing  [14]. LncRNA could localize in the nucleus or cytoplasm, and the function of lncRNA usually depends on subcellular localization [15]. Researchers have found that LncRNA was involved in multiple aspects of gene expression regulation including epigenetic, transcriptional and post-transcriptional modification. (1) LncRNA can act as an RNA decoy binding to transcription factors, thus interfering its binding to the promoter and regulating transcription [16]. (2) As a molecular sponge, adsorbing and separating miRNA from target mRNA, which affects the translational of mRNA [17]. (3) As a molecular scaffold, interacting with proteins to form the lncRNA-proteins complex, thereby regulating the protein activity or stability [18]. (4) Recruiting chromatin modifiers to reprogram chromatin [19]. (5) Binding with mRNA and affecting the translation, splicing, and stability of mRNA [20, 21] (Fig. 1). With the continuous research on lncRNA in recent years, lncRNA has been proved to be involved in tumor progression through a series of cell metabolism processes (Fig. 2), thus showing a unique advantage in tumor diagnosis, monitoring, prognosis and treatment [22]. LncRNAs can play an oncogenic or a tumor suppressive role, however, they are often dysregulated in cancers and participate in the occurrence of metabolic changes [23–25]. In this review, we mainly focus on the roles of lncRNAs in amino acid metabolism of tumor cells and discuss the pathways affected by lncRNAs in the process of cancer metabolism.

### Normal metabolism of amino acids

Metabolic reprogramming enables cancer cells to adapt to increased nutrient requirements and biosynthesis, in which changes in amino acid metabolism are an important part of the metabolic reprogramming event. Amino acids, which are metabolized into proteins and converted into hormones, neurotransmitters and other important nitrogen-containing active substances, are crucial for cell survival [26]. In the process of amino acid metabolism in normal cells, 1–2 % of proteins are degraded every day, mostly in skeletal muscle and most of the amino acids produced by protein degradation are reused to synthesize new proteins. Intracellular protein degradation is completed by a series of enzymatic reactions [27]. Eukaryotic cells participate in the degradation of protein mainly through two pathways: (1) ATP-independent pathway in lysosomes. (2) ATP-dependent pathway in the proteasome, namely the ubiquitin-involved protein degradation process [28]. In humans, the twenty amino acids that make up proteins are usually divided into essential and non-essential amino acids. Essential amino acids are those whose carbon skeleton cannot be de novo synthesized, including isoleucine (Ile), leucine (Leu), methionine (Met), valine (Val), phenylalanine (Phe), tryptophan (Trp), histidine (His), threonine (Thr) and lysine (Lys). Semi-essential amino acids are those that can be synthesized de novo but not in sufficient quantities to maintain the normal metabolic level. Therefore, dietary supplementation is usually required. They include arginine (Arg), cysteine (Cys), glycine (Gly), glutamine (Gln), proline (Pro) and tyrosine (Tyr). Five other amino acids are considered dispensable because they are easily synthesized in vivo, including alanine (Ala), aspartic acid (Asp), asparagine (Asn), glutamate (Glu) and serine (Ser) [29]. Glutamine is the most abundant amino acid in the circulation and is second only to glucose in the metabolism of tumor cells [30]. Although glutamine is a kind of conditionally essential amino acid in vivo, it has been observed that in lots of cancers, the dependence on glutamine has unique to some cancer cells [31, 32].

### Amino acids are indispensable for tumor cell metabolism

Mounting evidence shows that the rapid proliferation of cancer cells depends on higher demand for amino acids. A variety of amino acids play a vital function in the metabolism of tumor cells: Serine and glycine, as the basic precursors for the synthesis of proteins, nucleic acids and lipids, need more consumption to meet the rapid proliferation of tumor cells. Moreover, the biosynthesis of serine and glycine affects cellular antioxidative capability and also promotes tumor growth [33]; The c-MYC oncogene activates the expression of glutaminase (GLS1/GLS2) and glutamine metabolism in cancer cells, and glutamine can be converted to glutamate even in hypoxia [34]. More consumed glutamine for mitochondrial energy production is a feature of multiple cancer cell metabolism; arginine participates in the urea cycle, in which argininosuccinate synthetase (ASS) catalyzes citrulline and aspartic acid to produce argininosuccinate. Subsequently, argininosuccinate is cleaved to produce arginine. In malignant melanoma and hepatocellular carcinoma, ASS deficiency leads to a failure in arginine synthesis, and arginine deficiency interferes with the growth of cancer cells, which is used for the treatment of advanced malignant tumors [35]. Furthermore, tumor cells also preferentially uptake branched chain amino acids (BCAAs) as nutrients. The three BCAAs are valine, leucine and isoleucine. BCAA metabolism can meet some inherent requirements in the process of cancer proliferation, such as providing nitrogen for de novo nucleotide synthesis, participating in the activation of signaling pathways and influencing the expression of many crucial metabolite-derived co-factors [36]. In melanoma, for example, when leucine is deprived, hyperactivation of RAS-MEK signaling fails to inhibit mTOR, thereby triggering apoptosis of human melanoma cells [37].

### Glutamine metabolism: the essential part of amino acid metabolism

**Fig. 3:**
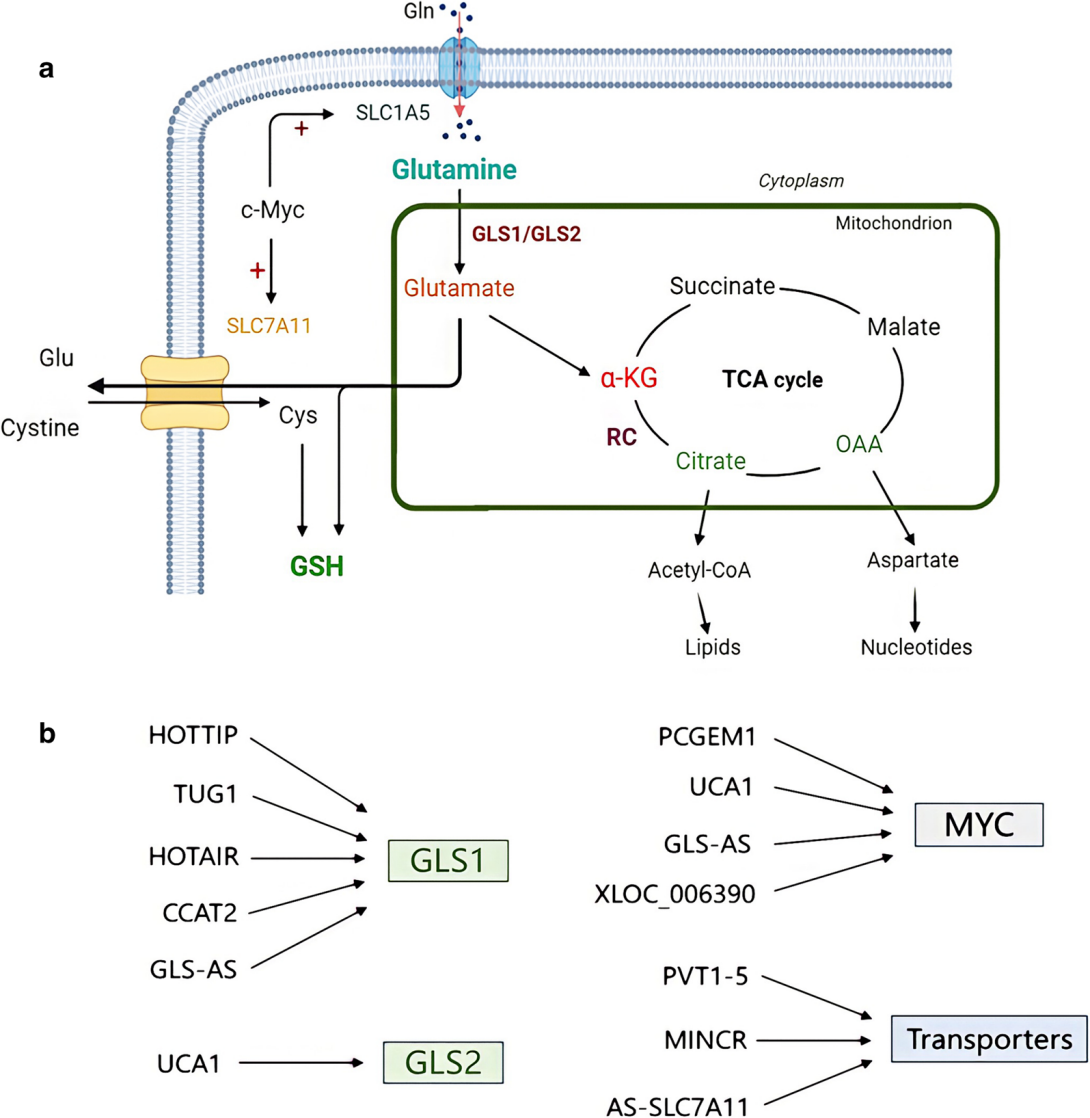
**a** SLC1A5 transports glutamine into cells and is converted to glutamate by glutaminase, which participates in the synthesis of GSH together with intracellular Cys. SLC7A11 exchanges extracellular cystine and intracellular glutamate, and c-myc participates in the positive regulation of SLC1A5 and SLC7A11. Glutamate is converted into α-KG by L-glutamate dehydrogenase or aminotransferases, which participates in the TCA cycle. α-KG participates in the reductive carboxylation process to produce citrate, which used for the synthesis of acetyl-CoA and lipids, aspartate produced by oxaloacetic acid transamination is a necessary substance for nucleotide synthesis. **b** LncRNAs which are involved in regulating amino acid metabolism

Glutamine is transported into cells by solute carrier family 1 neutral amino acid transporter member 5 (SLC1A5, also known as ASCT2), and high levels of glutamine in the blood serve as a source of nitrogen and carbon for biosynthesis. Intracellular glutamine could be catalyzed by mitochondrial glutaminase to produce glutamate and ammonium ions. Glutamate is also a precursor to glutathione (GSH), which is a major cellular antioxidant to help maintain normal immune system function [38–40]. Glutamate could be converted into α-KG (α-Ketoglutarate), the intermediate product of the tricarboxylic acid (TCA) cycle, by transamination and oxidative deamination. The oxidative deamination of glutamate is catalyzed by GDH (glutamate dehydrogenase) [41]. Moreover, α-KG is involved in reductive carboxylation and reverse catalysis to produce citrate used for the synthesis of acetyl-CoA and lipids [42]. Oxaloacetic acid (OAA), an intermediate of the TCA cycle, is converted into aspartic acid by transamination, which is used for the synthesis of purine and pyrimidine nucleotides (Fig. 3). At the same time, glutamine can promote the synthesis of UDP-N-acetylglucosamine (UDP-GlcNAc), which plays a key role in protein folding and transport. The lack of glutamine will lead to faulty protein folding and endoplasmic reticulum stress response [43].

#### LncRNAs and glutaminase

Glutamine can be catalyzed by glutaminase to produce glutamate, which is the first step of glutamine metabolism. There are two genes encoding glutaminase in mammals, namely GLS and GLS2, which show different structures and are specifically expressed in different tissues [44, 45]. Recent studies have shown that GLS is highly expressed in some malignant tumors and knockdown of GLS significantly reduces the invasion and proliferation of cancer cells, indicating the cancer-promoting role of GLS [46, 47]. GLS2 is mainly expressed in the liver and brain [48]. In some tissues, GLS2 is a target of p53 and mediates the tumor-suppressing role of p53 in cancer cells [49]. LncRNA CCAT2 (Colon Cancer Associated Transcript 2), which is located at the 8q24 amplicon of cancer risk-associated rs6983267 SNP was reported to promote the glycolysis as well as glutamine metabolism in a variety of cancers [50, 51]. The interaction between CCAT2 and CFIm complex regulates the alternative splicing of GLS by selecting the Poly-A site of GLS pre-mRNA intron 14 and induces the production of two alternative splicing isoforms, respectively KGA(glutaminase kidney isoform) and GAC(glutaminase isoform C)  [51]. Although these two isoforms have the same active site, GAC has higher catalytic activity than KGA. GAC plays a key role in mitochondrial glutamine metabolism of cancer cells, promoting cell proliferation and metastasis in vivo [52].

#### The way lncRNAs regulate glutamine metabolism

Previous studies have shown that miRNA can interact with specific lncRNA and degrade lncRNA at the post-transcriptional level [53]. LncRNA HOTTIP is an important oncogene of hepatocellular carcinoma (HCC). It was reported that HOTTIP was involved in GLS1-mediated glutamine metabolism in HCC, and HOTTIP overexpression could improve GLS1 expression level to enhance glutamine metabolism. HOTTIP was identified as the target of miR-192 and miR-204, which would inhibit HOTTIP expression through the Argonaute2-mediated RNA interference pathway, thereby inhibiting the proliferation of the cancer cells. GLS1, as a downstream gene of the miR-192/204-HOTTIP axis, is critical in the progression of hepatocellular carcinoma and glutamine metabolism [54].

LncRNA can interact with miRNA as an endogenous competitive RNA (ceRNA), and miRNA participates in the regulation of target gene expression by binding with the 3’ UTR of target mRNA [55]. LncRNA TUG1 has been reported to promote glutamine metabolism by inhibiting miR-145 in intrahepatic cholangiocarcinoma (ICC). Sirt3, as a direct target of miR-145, has been confirmed to activate GDH in the mitochondrial matrix by deacetylation, thus positively regulating the expression of GDH [56]. LncRNA TUG1, acting as a ceRNA ‘sponges’ miR-145, increased the level of Sirt3 and GDH. The knockdown of TUG1 or overexpression of miR-145 resulted in a significant reduction in intracellular glutamine consumption, reduced proliferation and migration of ICC cells, and inhibited tumor development. At the same time, glutamate participates in GDH-mediated oxidative deamination which results in the reduction of α- KG  [57]. LncRNA HOX transcript antisense intergenic RNA(HOTAIR) has a similar effect. In glioma cells, the expression of lncRNA HOTAIR is abnormally increased. Existing studies have shown that lncRNA HOTAIR as a ceRNA ‘sponged’ miR-126-5p and promotes glutamine metabolism in glioma. The miR-126-5p was reported to play an inhibitory role in both lung cancer and gastric cancer [58, 59]. While in glioma, GLS was confirmed to be the direct target of miR-126-5p, and miR-126-5p significantly reduced the expression of GLS in mRNA and protein levels. LncRNA HOTAIR regulated the expression of GLS through the miR-126/GLS pathway, thus changing the glutamine metabolism process of glioma and promoting the development of the tumor. Glutamate was also the precursor of GSH, while miR-126-5p was negatively correlated with GSH level [60].

#### LncRNAs mediate the antioxidant defense in cancer metabolism

Glutamine metabolism is of great significance for maintaining the redox balance of tumor cells and the level of ROS (reactive oxygen species). GLS2 inhibits the production of ROS and mediates the antioxidant defense function of cells [61]. Glutamine can be catalyzed by GLS2 to produce glutamate, which participates in the synthesis of glutathione (GSH) in vivo. The glutathione-centered redox system is involved in the occurrence of a series of signal pathways, including the elimination of ROS, protein synthesis and cell oxidative defense functions. GSH is the most important intracellular antioxidant molecule, protecting cells from apoptosis induced by oxidative stress [62, 63]. Glutathione mainly exists in the form of reduced glutathione (GSH) and oxidized glutathione (GSSG). Under physiological conditions, GSH is the main existing form, accounting for about 99 % [64]. The ratio of GSH/GSSG reflects the redox state of cells, the lack of glutamine during cell metabolism will eliminate the effect of GLS2 on the increase of GSH levels, indicating that glutamine metabolism plays a critical role in maintaining tumor cell redox balance and ROS levels  [65].

It was reported that lncRNA UCA1 participated in the malignant progression, drug resistance and metabolism reprogramming of bladder cancer [66–69]. In bladder cancer cells, UCA1 regulates the glutamine metabolism and antioxidant defense by inhibiting miR-16, which targets GLS2 for translational inhibiting and reduced GLS2 expression. This suggests a positive role of UCA1 in reducing ROS and sustaining the redox balance of cancer cells. Notably, the mRNA level of UCA1 and GLS2 are positively correlated, and the expression of GLS2 is negatively correlated to the miR-16 in bladder cancer.

#### The interplay between lncRNAs and MYC

The MYC proto-oncogene family includes c-Myc, N-Myc, L-Myc, which are involved in the occurrence of various human tumors [70]. Cancer cells maintain their rapid proliferation and metastasis through metabolic reprogramming. Studies on PI3K/AKT/mTOR signaling pathway showed that cancer cells were strongly addicted to glucose and other nutrients such as amino acids. Glutamine, as one of the major energy substrate of cancer cells, could also easily lead to addiction [71]. Metabolic reprogramming induced by MYC leads to glutamine addiction, its high expression can induce the glutamine transporter, glutaminase and lactate dehydrogenase A (LDH-A) expression. Myc is a major regulator of glutamine metabolism [72]. Cancer cells accelerate mitochondrial glutaminolysis by Myc, which provides cells with fast-generating NADPH [73]. NADPH participates in many metabolic reactions, such as the synthesis of fatty acids, cholesterol and non-essential amino acids, and plays a key role in maintaining the reduction state of GSH [74].

As we all know, the androgen receptor (AR) signaling plays an important role in the progression of prostate cancer [75]. LncRNA PCGEM1 (prostate cancer gene expression marker 1) is an androgen-induced prostate-specific lncRNA [76], which has been confirmed to regulate the metabolism including the tricarboxylic acid cycle, glutamine metabolism and pentose phosphate pathway of prostate cancer by activating c-Myc. C-Myc recruits PCGEM1 to the promoter of its target genes, which promotes chromatin recruitment and enhances its transactivation activity. When endogenous PCGEM1 is knocked down, the activity of c-Myc will decrease, indicating that PCGEM1 plays a metabolic regulation role as a co-activator of c-Myc in prostate cancer cells. This is also the first report that lncRNA binds with c-Myc and acts as a co-activator to regulate metabolic reprogramming [77]. In esophageal squamous cell carcinoma (ESCC), the Wnt/β-catenin signaling pathway is highly correlated with the progression of ESCC, and its abnormal activation can lead to the occurrence of a variety of cancers [78]. While c-Myc, as a target gene of the Wnt/β-catenin signaling pathway, is involved in regulating ESCC cell cycle distribution and promoting tumor progression. The overexpression of UCA1 reduced the level of c-Myc and the β-catenin protein in the nucleus, and the proliferation and invasion of ESCC cells were obviously inhibited [79]. On the other hand, pancreatic cancer (PC) is a group of malignant tumors that mainly originated from the pancreatic ductal epithelium and acinar cells, which is extremely malignant and progresses rapidly  [80]. Recent studies have found that antisense lncRNA of glutaminase (GLS-AS) is involved in the pathogenesis of pancreatic cancer by mediating the mutual feedback of Myc and GLS in the tumor nutrients stress microenvironment. Tumor nutrients stress microenvironment is a critical factor for the downregulation of GLS-AS in pancreatic cancer, and GLS is the key target of GLS-AS. GLS-AS forms double-stranded RNA with GLS pre-mRNA and inhibits GLS expression at the post-transcriptional level. The promoter region of GLS-AS contains the binding site of Myc. As a multifunctional transcription factor, Myc down-regulates the expression of GLS-AS by inhibiting its transcriptional activity [81]. C-Myc is involved in regulating a variety of signaling pathways in cancer cells, and recent studies have shown that c-Myc participates in the regulation of glutamine metabolism by mediating the transcription of GDH. LncRNA XLOC_006390 was also found to promote pancreatic cancer through regulating amino acid metabolism. XLOC_006390 promotes the stability of c-Myc by preventing its ubiquitination and subsequently upregulates GDH. GDH was confirmed to be generally up-regulated in cancer, and knockdown of GDH in cancer cells can significantly attenuate the glutaminolysis rate [82]. As GDH is closely related to pancreatic cancer progression, reduce the expression of GDH via the XLOC_006390/c-Myc/GDH signal axis, leading to the lower glutamine metabolism level, thus may providing a new vision for pancreatic cancer therapy [83].

### LncRNAs mediate amino acid transporters’ function in cancer

The proliferation of tumor cells is characterized by uncontrolled and rapid division. Amino acids, as a class of major nutrients, are very important for the growth of tumor cells [84]. Due to excessive nutrient requirements, some amino acid transporters are up-regulated in the development of cancer. Four amino acid transporters have been found to be highly expressed in cancer, namely SLC7A5, SLC7A11, SLC1A5 and SLC6A14. SLC7A5 is also referred to as the LAT1 (L-Amino acid transporter 1), with a high affinity to the branched-chain and neutral amino acid. It mediates a sodium-independent mandatory exchange and this exchange mechanism allows a large number of neutral amino acids to be balanced on the membrane [85]. The promoter of SLC7A5 has a typical binding site with C-Myc, and the high expression of C-Myc often leads to an increase of SLC7A5 expression level in cancer cells [86]. It was reported that SLC7A5 was closely related to cell proliferation in lung cancer. SLC7A5 has been proved to be the direct target of miR-126 [87], which significantly inhibits the transport of other amino acids such as leucine, thus affecting the amino acid metabolism and the activation of the mTOR signal  [88, 89]. The involvement of leucine in mTOR signal activation has been widely recognized [90]. Several lncRNAs were reported to regulate the amino acids metabolism through miR-126. For example, lncRNA PVT1-5 (Plasmacytoma variant translocation 1–5) was reported to be up-regulated and promoted the progression of lung cancer through sponging miR-126 [91]. Moreover, in non-small cell lung cancer (NSCLC), MYC induced long noncoding RNA (MINCR) is significantly up-regulated and promoted the proliferation and migration of NSCLC cells through targeting miR-126 [92].

Cysteine, as a rate-limiting amino acid for GSH synthesis, whose level affects GSH balance in cells, is considerable for the sustaining of redox balance of cells. SLC7A11, the major transporter for the exchange of extracellular cysteine and intracellular glutamate, protects cancer cells from apoptosis and promoting tumor development by improving the synthesis of GSH [93]. Antisense lncRNA AS-SLC7A11 is significantly reduced in epithelial ovarian cancer (EOC) and has been proved to inhibit SLC7A11 expression in ovarian cancer. The knockdown of AS-SLC7A11 increases the expression of SLC7A11 and improves the proliferation and viability of ovarian cancer cells [94].

### Other mechanisms of lncRNAs in amino acid metabolism

The role of lncRNAs in cancer is diverse, recent studies have shown that polypeptides encoded by lncRNA can also suppress cancer in amino acid metabolism  [95]. LncRNA HOXB-AS3 can encode a conserved peptide containing 53 amino acids. This peptide inhibits amino acid metabolism and glycolysis to slow the progression of colon cancer [96]. As we know, the methionine cycle elucidates the metabolism of sulfur-containing amino acids in vivo. Methionine adenosyltransferases (MAT) catalyze the methionine cycle to produce S-adenosylmethionine (SAMe). MAT has two coding genes in vivo, respectively MAT1A and MAT2A, and in the liver, their regulation modes for the SAMe are completely opposite: MAT1A upregulates the concentration of the SAMe, while MAT2A downregulates the SAMe [97, 98]. The methyl in SAMe is called active methyl, which makes SAMe to be the most important direct donor of methyl in vivo [99]. In hepatocellular carcinoma (HCC), lncRNA SNHG6, as a molecular sponge of miR-1297, is involved in regulating genome-wide methylation levels. MiR-1297 directly binds to the 3 ‘UTR of the MAT2A mRNA, leading to its translational inhibition. SNHG6 upregulates the expression of MAT2A, thereby negatively regulating the concentration of SAMe in cells, leading to significant genome-wide hypomethylation of hepatocellular carcinoma, which is a major feature of tumor genesis [100].

##  Conclusions

LncRNA in malignant tumors is widely involved in the process of metabolism  [101]. Besides involved in the regulation of glycolysis and lipid metabolism [102], lncRNA was also found to be involved in the process of amino acid metabolism: regulate the action mode of amino acid transporters, leading to the lack of several amino acid types; as a ceRNA to interact with miRNA involved in regulating glutamine metabolism; control alternative splicing of glutaminase to regulate metabolic processes in vivo; reduce intracellular ROS level so as to protect the function of the mitochondrion and regulate antioxidant defense in cells; encode some peptides to play a role in the anticancer properties, which can enlighten some potential ideas for treatment; play a unique role in the regulation of signaling pathways in Myc-driven cancers. In this review, we highlighted the characteristics of lncRNAs in regulating cancer amino acid metabolism, and the change of glutamine metabolism played a vital role in the process of oncogenesis [103]. Tumor cells seem to be very dependent on glutamine so that we can reasonably infer that drugs act on glutamine metabolism can play an unexpected role in the process of cancer development. The rediscovery on the effect of glutamine in tumor cells may provide us with promising clinical treatment. Besides, the metabolic mechanism of lncRNA in cancer also needs to further elucidate, which will be a great help to find new biomarkers and therapeutic targets in cancer treatment. There are many other lncRNAs related to cancer cell metabolism, whose structures and functions are not clear to us. Given that the research on lncRNAs is still at the preliminary stage, it seems promising to discover novel lncRNAs and develop lncRNA-based targeted therapeutic strategies. The study afterward will be full of challenges and opportunities.

## Data Availability

The material supporting the conclusion of this review has been included within the article.

## Abbreviations

lncRNAs: Long non-coding RNAs
ncRNA: Noncoding RNA
ceRNA: Endogenous competitive RNA
GLS: Glutaminase
KGA: Glutaminase kidney isoform
GAC: Glutaminase isoform C
GSH: Glutathione or reduced glutathione
GSSG: Oxidized glutathione
α-KG: α-Ketoglutarate
ASS: Argininosuccinate synthetase
GDH: Glutamate dehydrogenase
ROS: Reactive oxygen species
LDH-A: Lactate dehydrogenase A
TCA cycle: Tricarboxylic acid cycle
MAT: Methionine adenosyltransferases
Ile: Isoleucine
Leu: Leucine
Met: Methionine
Val: Valine
Phe: Phenylalanine
Trp: Tryptophan
His: Histidine
Thr: Threonine
Lys: Lysine
Arg: Arginine
Cys: Cysteine
Gly: Glycine
Gln: Glutamine
Pro: Proline
Tyr: Tyrosine
Ala: Alanine
Asp: Aspartic acid
Asn: Asparagine
Glu: Glutamate
Ser: Serine
OAA: Oxaloacetic acid
BCAAs: Branched chain amino acids
SLC1A5: Solute carrier family 1 neutral amino acid transporter member 5
LAT1: L-Amino acid transporter 1
UDP-GlcNAc: UDP-N-acetylglucosamine
AR: Androgen receptor
SAMe: S-adenosylmethionine
CCAT2: Colon Cancer Associated Transcript 2
HOTAIR: HOX transcript antisense intergenic RNA
PVT1-5: Plasmacytoma variant translocation 1-5
MINCR: MYC induced long noncoding RNA
PCGEM1: Prostate cancer gene expression marker 1
GLS-AS: Antisense lncRNA of glutaminase
HCC: Hepatocellular carcinoma
ICC: Intrahepatic cholangiocarcinoma
PC: Pancreatic cancer
NSCLC: Non-small cell lung cancer
ESCC: Esophageal squamous cell carcinoma
EOC: Epithelial ovarian cancer
